# Investigating circulating expression profile for *H19*, *MEG3*, *and MIAT* long noncoding RNAs with *miR-135a* and *miR-29a* in chronic kidney disease and renal hemodialysis patients: interrelations with serum sclerostin

**DOI:** 10.3389/fmolb.2025.1703108

**Published:** 2026-01-23

**Authors:** Marwa A. Dahpy, Marwa K. Khairallah, Rania Naguib, Mona A. Khalil, Salwa Seif Eldin, Marwa A. Sabet, Amira A. Kamel

**Affiliations:** 1 Department of Medical Biochemistry and Molecular Biology, Faculty of Medicine, Assiut University, Assiut, Egypt; 2 Department of Medical Biochemistry and Molecular Biology, Armed Forces College of Medicine (AFCM), Cairo, Egypt; 3 Department of Internal Medicine, Faculty of Medicine, Assiut University, Assiut, Egypt; 4 Department of Internal Medicine, College of Medicine, Princess Nourah bint Abdulrahman University, Riyadh, Saudi Arabia; 5 Department of Biochemistry and Molecular Biology, Faculty of Medicine, Al-Azhar, University, Cairo, Egypt; 6 Department of Basic Medical Sciences, College of Medicine, Princess Nourah Bint Abdulrahman University, Riyadh, Saudi Arabia; 7 Department of Microbiology and Immunology, Faculty of Pharmacy, Sphinx University, New-Assiut, Egypt

**Keywords:** chronic kidney disease, hemodialysis, lncRNA H19, lncRNA MIAT, LncRNAMEG3, miR-135a, miR-29a, quantitative polymerase chain reaction

## Abstract

**Background and aim:**

Chronic kidney disease (CKD) incorporates a variety of progressive kidney function declines, ranging from mild impairment to end-stage renal disease (ESRD). As a global public health problem, early detection and monitoring remain critical for improving patient outcomes. This study aimed to evaluate the potential diagnostic significance and interrelationships of serum sclerostin, *lncRNA H19*, *lncRNA MEG3*, *lncRNA MIAT*, *miR-135a*, and *miR-29a* in patients with CKD and those undergoing hemodialysis.

**Methods:**

A total of 150 participants were enrolled in this present case–control study: 50 hemodialysis patients, 50 CKD patients, and 50 healthy controls. Circulating serum sclerostin levels were measured using ELISA. Real-time quantitative PCR (RT-qPCR) was used to measure the circulating levels of lncRNAs (*H19*, *MEG3*, and *MIAT*) and microRNAs (*miR-135a* and *miR-29a*). A spectrophotometer was used to determine the levels of calcium, creatinine, urea, and phosphorus in the blood.

**Results:**

Both the CKD and hemodialysis groups displayed significantly elevated levels of all studied biomarkers compared with controls. However, the highest levels of sclerostin, lncRNAs (*H19*, *MEG3*, and *MIAT*), and microRNAs (*miR-135a* and *miR-29a*) were found in the hemodialysis group. LncRNA *MIAT*, *miR-135a*, and *miR-29a* showed high sensitivity and specificity in distinguishing CKD patients from healthy controls. Additionally, *H19*, *MEG3*, and *miR-29a* displayed strong differentiating ability between CKD and hemodialysis patients. All studied biomarkers exhibited strong positive correlations. Predictive modeling identified *lncRNAs H19*, *MEG3*, and *MIAT* as significant predictors of hemodialysis status, while sclerostin, *MEG3*, and *MIAT* were the strongest predictors of CKD.

**Conclusion:**

Serum sclerostin, lncRNAs (*H19*, *MEG3*, and *MIAT*), and microRNAs (*miR-135a* and *miR-29a*) are significantly interrelated and may serve as promising non-invasive molecular biomarkers for early detection and monitoring of CKD and hemodialysis patients.

## 1 Introduction

Chronic kidney disease (CKD) is a global health challenge characterized by a gradual decline in renal function and is associated with substantial morbidity and mortality (Maringhini and Zoccali, 2024). A hallmark of CKD progression is renal fibrosis, a pathological process involving excessive accumulation of extracellular matrix components that ultimately leads to irreversible structural damage and functional impairment of the kidneys (Reiss et al., 2024). To improve the early diagnosis and design specific treatment methods, it is essential to understand the molecular processes that mediate renal fibrosis.

One of the novel regulating factors in CKD is sclerostin, a glycoprotein that is a product of the *SOST* gene. Sclerostin is traditionally considered an inhibitor of Wnt/b-catenin signaling and an osteoblast activity regulator (Vasiliadis et al., 2022). Recent results indicate that the levels of circulating sclerostin increase with deteriorated kidney performance, and research indicates correlations between serum sclerostin and eGFR and its role in CKD-mineral bone disorder (CKD-MBD) (Izzo et al., 2024; Simic et al., 2024). Furthermore, clinical evidence shows that vascular calcification, arterial stiffness, and negative renal outcomes are all associated with high levels of sclerostin in CKD patients (Afsar et al., 2024). Mechanistically, it has recently been indicated that sclerostin may play a direct role in renal fibrosis by regulating interferon-stimulated gene (ISG20)-mediated pathways (Han et al., 2025). Although this continues to be compiled evidence, the exact correlation between sclerostin and the molecular determinants of renal fibrosis has not been fully comprehended.

At the same time, non-coding RNAs (ncRNAs) have emerged as key regulators of gene expression during renal pathophysiology. One of the initial lncRNAs to be identified is long non-coding RNA *H19*, which plays extremely significant roles in cancer and tissue remodeling (Lin et al., 2020). *H19* dysregulation plays a significant role in renal fibrosis and, therefore, its functional role in causing kidney injury (Liao et al., 2023). Maternally expressed gene 3 (*MEG3)* is another lncRNA that is associated with fibrosis. *MEG3* has an impact on most cellular pathways, including the *TGF-b* signaling cascade, which is a major component of fibrogenesis. The reduced or altered expression of *MEG3* is associated with the occurrence of renal fibrosis, suggesting that it could be a biomarker and therapeutic target (Hussain et al., 2023). Similarly, apoptosis, inflammation, endothelial dysfunction, and fibrotic processes in kidney disease are associated with the dysregulation of lncRNA *MIAT* (Motshwari et al., 2022; Zeinelabdeen et al., 2024). *MIAT* is implicated in complex regulatory pathways that regulate the response to renal cell injuries and renal cell homeostasis. It achieves this through the interaction with microRNA (miRNA), such as *miR-29a* (Zhan et al., 2023).

miRNAs are critical post-transcriptional regulators that play an important role in kidney development and disease. It is known that *miR-29* family members regulate the extracellular matrix turnover and fibrosis, and therefore, they are associated with the development of CKD and can be used as promising diagnostic and therapeutic solutions (Horita et al., 2021; Sun et al., 2023; Sasso et al., 2024). Furthermore, *miR-135a* also regulates genes associated with apoptosis and essential signaling pathways, including Wnt/b-catenin, thus confirming its role in renal inflammation and fibrogenesis (Kadkhoda et al., 2022).

The convergence of sclerostin and the ncRNAs (*H19*, *MEG3*, *MIAT*, *miR-29a*, *and miR-135a*) that were investigated in this study on the key pathways central to the pathogenesis of CKD (Wnt/b-catenin signaling, extracellular-matrix control, inflammation, and mineral metabolism) suggests that they may collectively represent an integrated molecular signature of CKD progression. The Wnt-inhibitory activity of sclerostin is also in line with the regulatory activity of *miR-135a*, whereas *miR-29a* has a direct effect on matrix deposition, opposing the pro-fibrotic activity of *H19*, *MEG3*, *and MIAT*.

Furthermore, these specified ncRNAs exhibit consistent differences in expression in CKD based on both experimental findings and publicly available transcriptomic data (Yang et al., 2025; Ramanathan et al., 2024; Dong et al., 2022; Zhang, et al.,2020; Che et al., 2019), and they can be reliably detected in circulation (Alvarez and Distefano, 2013). Collectively, such a panel offers complementary biological coverage, supports its use as an integrated set of biomarkers for CKD.

Therefore, their simultaneous assessment in a coordinated panel can be considered a more global report of the disrupted bone–vascular–kidney axis and fibrogenic microenvironment of CKD, as opposed to individual markers.

Since they are related to renal fibrosis, inflammation, vascular remodeling, and mineral metabolism, the lncRNAs *H19*, *MEG3*, and *MIAT*, combined with *miR-29a* and *miR-135a*, are promising biomarkers in the circulation of CKD. Together with the concomitant dysregulation of serum sclerostin, these indicators can provide new data on the perturbed bone–vascular–kidney axis of CKD. Nevertheless, although there is increasing evidence on the respective roles, the relationships between these ncRNAs and sclerostin, along with their synergistic effect on CKD progression, are not well understood. To address this issue, current research compares the expression levels of these markers in CKD patients, hemodialysis patients, and normal controls to understand their possible role in renal fibrosis and assess them as the biomarkers of disease pathogenesis and progression.

## 2 Subjects and methods

### 2.1 Study subjects

The study was carried out at the departments of Medical Biochemistry and Internal Medicine, Faculty of Medicine, Assiut University. The Institutional Review Board approved the study. One hundred and fifty adult participants were recruited in this case–control study. Prior to the study, all contributors provided their written informed consent. The participants were recruited into their clinical groups. Three groups were included: 50 healthy adults served as controls in group I, 50 CKD patients as group II, and 50 hemodialysis patients as group III. Group III patients with end-stage renal disease (ESRD) were placed on maintenance hemodialysis in the Internal Medicine Department’s hemodialysis unit three times a week for 4 h each session (via arteriovenous fistulas). All patients and controls were matched in terms of age and sex.

Patients with stable and well-documented comorbidities, such as diabetes mellitus (DM) and hypertension (HTN), were not excluded to reflect real-world clinical presentation. A representative evaluation of circulating non-coding RNA profiles in typical CKD and hemodialysis patients was made possible by the inclusion of these conditions, which are highly prevalent in CKD populations.

The exclusion criteria included pregnant women; patients with infectious diseases; those using corticosteroids or anti-inflammatory medications; those with malignancies, inflammatory diseases, congenital renal abnormalities, systemic autoimmune diseases, acute kidney injury, decompensated liver disease, recent major surgery, or current use of immunosuppressive therapies. Patients with uncontrolled diabetes (e.g., markedly elevated HbA1c) or uncontrolled hypertension were excluded to reduce confounding effects related to severe metabolic or hemodynamic instability.

### 2.2 Bioinformatics databases

We investigated a selected panel of RNAs specific to renal diseases; the selected panel is composed of lncRNA*H19*, lncRNA*MEG3*, and lncRNA *MIAT*, together with *miR-135a* and *miR-29a*. We searched using the following genetic databases: the Human microRNA Disease Database Version 4.0 (HMDD V 4.) (http://www.cuilab.cn/hmdd) and the MIRWALK database (http://mirwalk.umm.uni-heidelberg.de). The sequence identity and annotation of the used parameters were verified using the NCBI Gene database. Inclusion in the molecular panel is based on its previously reported involvement in renal inflammation and fibrosis. Validation of the chosen RNAs was performed using real-time quantitative PCR in whole blood from patients with CKD and ESRD undergoing hemodialysis, compared with control groups.

### 2.3 Blood sample collection

Five milliliters of antecubital venous blood were collected from each participant. Three milliliters of blood were collected in plain tubes and allowed to coagulate, while 2 mL of whole blood were collected in EDTA tubes and stored at −80 °C until molecular analysis of lncRNAs and miRs. The plain tubes were centrifuged at 3,000 rpm for 20 min at 4 °C to collect the serum, which was then stored at −20 °C for ELISA and spectrophotometer analysis. All samples were labeled only with coded identifiers, and decoding was performed after completion of all laboratory measurements. In addition, all laboratory personnel were blinded to participants’ clinical group assignments.

### 2.4 Estimation of sclerostin through ELISA

Serum sclerostin levels were quantified using a commercially available human sclerostin ELISA kit, catalog no #95081, which is provided by Glory Science Co., Ltd. All steps were performed according to the manufacturer’s instructions. Serum samples were diluted 1:5 using the supplied sample diluent. Standards and diluted samples were incubated for 30 min at 37 °C, followed by plate washing (five cycles) with the supplied 1:30 diluted wash buffer. Absorbance was measured using a microplate reader at 450 nm within 15 min. Concentrations were derived from a standard curve generated from supplied calibrators.

### 2.5 Estimation of circulating expression levels of lncRNAs *H19*, *MEG3*, and *MIAT*, along with *miR-135a* and *miR-29a*, using qRT-PCR

Total RNAs were extracted using the PureLink™ RNA Mini Kit (Invitrogen, Catalog No. 12183020), and the NanoDrop spectrophotometer was used to measure their concentration. The polyadenylate polymerase enzyme was added particularly after the final steps of miRNA extraction.

A total of 1 µg of RNA was used as input for each cDNA synthesis reaction, and all RNA samples met purity criteria (A260/280 between 1.8 and 2.1). A Thermo Scientific High-Capacity cDNA Reverse Transcription Kit (catalog no. 4368814) was used for cDNA reverse transcription experiments. Each cDNA reaction was performed in a total volume of 20 µL according to the manufacturer’s instructions, and the resulting cDNA was diluted 1:5 prior to qRT-PCR to ensure optimal amplification efficiency. cDNA was amplified using a PCR thermal cycler under the following conditions: initial denaturation for 2 min at 95 °C, followed by 40 cycles of denaturation for 10 s at 95 °C, annealing for 30 s at 56 °C, and an extension step for 30 s at 72 °C.

Quantitative real-time PCR: Each qRT-PCR reaction was performed in a final volume of 20 μL, containing 2 µL diluted cDNA, 10 µL SYBR^TM^ Green Master Mix obtained from Thermo Fisher Scientific [Catalog No. 4309155, Life Technologies, Applied biosystems, Applied Biosystems^TM^, USA], 0.5 µL of each forward and reverse primer (10 pmol/μL), and nuclease-free water. All reactions were run in duplicate to ensure reproducibility. As internal references, glyceraldehyde 3-phosphate dehydrogenase (GAPDH) and U6 were used. Applied Biosystems StepOne Plus™ software was utilized. Each gene’s expression was represented by a fold-change relative expression level using the comparative 2^-ΔΔCT method (Livak and Schmittgen, 2001). The sequences of all of the primers used are shown in Table 1.

**TABLE 1:** Primer sequences used in a quantitative real-time polymerase chain reaction.

Gene	Primer sequence 5′ to 3′
LncRNA H19 NR_131223.2	Forward 5′CGTCCGGCCTTCCTGAACA 3′ Reverse 5′TTGAGCTGGGTAGCACCATTTCT 3′
LncRNA*MEG3* NR_046471.1	Forward 5′ GGGCTTCTGGAATGAGCATGCTACTG 3′ Reverse 5′ CATTCGAGGTCCCCTTCCCACGTAGGCATC 3′
LncRNA MIAT NR_185986.1	Forward 5′ GAGATTGGCGATGGTTGTGA Reverse 5′ CAGTGACGCTCCTTTGTTGAA
*miR-135a* Shi et al. (2018)	Forward 5′ TTTCAGCTGGGGAGTGATTG 3′ Reverse 5′ GCAATCTCTGTGAATGGGTCA 3′
*miR-29a* Zhang et al. (2019)	Forward 5′TAGCACCATTTGAAATCAGTTT -3′ Reverse 5′TGCGTGTCGTGGAGTC -3′
Housekeeping gene GAPDH NM_001289746.2	Forward 5′AGGTCGGTGTGAACGGATTTG -3′ Reverse 5′TGTAGACCATGTAGTTGAGGTCA -3′
Housekeeping gene U6 NR_104084.1	Forward 5′CTCGCTTCGGC AGCACA 3′ Reverse 5′AACGCTTCACGAATTT GCGT 3′

This table presents the forward and reverse primer sequences used for amplification of the studied long non-coding RNAs *(lncRNA H19*, *lncRNA MEG3*, and *lncRNA MIAT),* microRNAs *(miR-135a* and *miR-29a),* and housekeeping genes (*GAPDH for lncRNAs*; *U6 for microRNAs*).

### 2.6 Estimation of serum creatinine, urea, eGFR, calcium, and phosphorus

A spectrophotometer was used to quantify serum creatinine, urea, calcium, and phosphorus using Spectrum Diagnostics kits with catalog numbers 237001, 320001, 227001, and 294001, respectively. The eGFR for each subject was determined using the MDRD equation.

$$\begin{array}{ll} \text{eGFR }\left(\text{mL}/\min/1.73m2\right)= & 175\times {\left(Scr\right)}^{-1.154}\times {\left(age\right)}^{-0.203}\\ & \;\times \left(0.742\text{ if female}\right). \end{array}$$

### 2.7 Statistical analysis

All statistical analyses were performed using GraphPad Prism 8 software (San Diego, California, United States) and IBM SPSS software version 26 (SPSS Inc., Chicago, IL, United States). The percentage of missing data was low (<5%) and seemed to be random. For analyses that required complete cases, list-wise deletion was implemented; for descriptive statistics, available-case data were used. The study’s goals, data distribution, and possible confounding variables all influenced the selection of statistical techniques. For group comparisons, parametric or non-parametric tests were used after the Shapiro–Wilk test was applied to assess normality. Categorical variables were displayed as a number and %; continuous data were displayed as the mean ± SD. Categorical data were assessed using the chi-square (χ2) test. Statistical significance was established as *p* < 0.05.

Based on the normality of the data, the Mann–Whitney and Student’s t tests were used. A one-way analysis of variance (ANOVA) was used to examine group comparability. The Spearman correlation coefficient test was used to assess correlations between the studied parameters. Multivariate regression analysis was used to determine the most significant predictors for CKD and hemodialysis. Multicollinearity was assessed, skewed variables were log-transformed as needed, and data normality was evaluated to maintain model stability. The receiver operating curve (ROC) and cutoff values were calculated using the built-in functions of MedCalc Statistical Software (MedCalc Software Ltd., Ostend, Belgium) (using the following link: https://www.medcalc.org/en/manual/roc-curves.php.), which use the non-parametric DeLong method to compute the area under the ROC curve (AUC) and the resulting 95% confidence intervals (DeLong et al, 1988), and the Youden index was used to identify the point that maximized sensitivity and specificity and obtain optimal cutoff values for each biomarker (Youden, 1950). For each cutoff, sensitivity, specificity, and associated diagnostic performance metrics were reported.

## 3 Results

### 3.1 Demographics and clinical characteristics of the studied groups

Table 2 displays the baseline characteristics of CKD, hemodialysis patients, and controls. There were no discernible changes in BMI, age, or gender among the three groups (*p*-value >0.05), indicating sufficient participant matching. On the other hand, diverse biochemical alterations typical of the progression of CKD were noted; the serum levels of creatinine, urea, phosphorus, and WBCs were significantly higher in CKD patients than in controls, and they were also significantly higher in hemodialysis patients than in CKD patients. Conversely, the serum levels of calcium, hemoglobin, platelets, and eGFR in patients with CKD and hemodialysis were all significantly lower than those in the controls.

**TABLE 2:** Demographic, clinical, and biochemical characteristics of participants.

Variables/Groups	Controls (n = 50)	CKD (n = 50)	Hemodialysis (n = 50)	P1	P2	P3	P4
Age (years) Mean ± SD	49.46 ± 4.71	48.74 ± 5.63	50.60 ± 5.07	0.194	0.486	0.270	0.073
Sex Male n (%) Female n (%)	31 (62) 19 (38)	35 (70) 15 (30)	34 (68) 16 (32)	0.677	0.398	0.529	0.829
BMI (kg/m^2^) Mean ± SD	29.13 ± 6.67	27.09 ± 6.51	28.7 2 ± 4.04	0.187	0.084	0.725	0.167
Creatinine (mg/dL)	0.91 ± 0.14	3.53 ± 0.88	7.57 ± 1.01	**< 0.001**	**< 0.001**	**< 0.001**	**< 0.001**
Urea (mg/dL)	26.78 ± 5.68	99.28 ± 17.33	141.56 ± 34.31	**< 0.001**	**< 0.001**	**< 0.001**	**< 0.001**
eGFR(ml/min/1.73 m^2^)	102.84 ± 9.50	49.46 ± 8.36	18.26 ± 3.44	**< 0.001**	**< 0.001**	**< 0.001**	**< 0.001**
Calcium (mg/dL)	10.2 ± 0.89	9.13 ± 1.23	8.63 ± 0.78	**< 0.001**	**< 0.001**	**< 0.001**	**0.012**
Phosphorus (mg/dL)	3.52 ± 0.61	4.66 ± 0.95	6.09 ± 1.51	**< 0.001**	**< 0.001**	**< 0.001**	**< 0.001**
Hb (g/dL)	12.65 ± 4.5	11.41 ± 2.47	10.59 ± 1.77	**<0.001**	**0.002**	**< 0.001**	**0.041**
WBCs (×10^3^/cmm)	6.11 ± 1.77	7.33 ± 1.92	9.02 ± 3.47	**< 0.001**	**0.017**	**< 0.001**	**0.001**
Platelets (×10^3^/cmm)	270.80 ± 80.23	204.50 ± 53.03	177.62 ± 56.10	**< 0.001**	**< 0.001**	**< 0.001**	**0.038**

The data are presented as the mean ± SD or number (%) of patients. Abbreviations: CKD, chronic kidney disease; BMI, body mass index; Hb, hemoglobin; WBCs, white blood cells; SD, standard deviation, and a *p*-value <0.05 is considered statistically significant.

A one-way ANOVA, followed by the LSD *post hoc* test, was used for multiple comparisons. The chi-square test was used to compare proportions between groups. P1, comparison between groups; P2, comparison between controls and CKD; P3, comparison between controls and hemodialysis; P4, comparison between CKD and dialysis.

### 3.2 Serum sclerostin levels and circulating expression of lncRNAs and microRNAs

**FIGURE 1:**
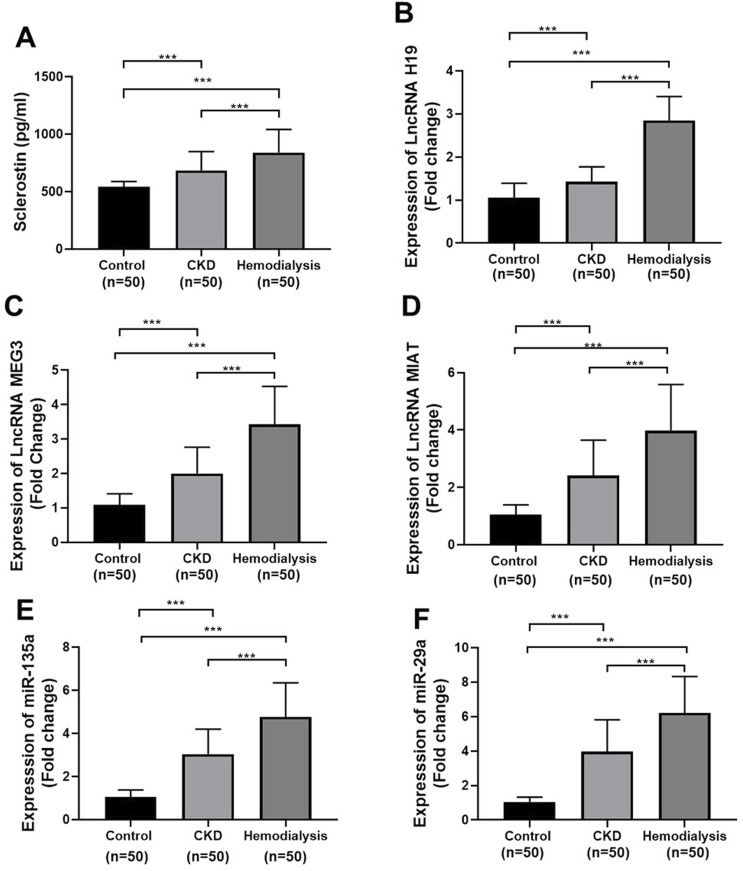
Serum levels of **(A)** sclerostin, and Expression of **(B)** lncRNA H19, **(C)** lncRNA MEG3, **(D)** lncRNA MIAT, **(E)** miR-135a, and **(F)** miR-29a in controls, CKD, and hemodialysis patients. Data are resented as the mean ± SD. Quantitative real-time PCR (qRT-PCR) data are presented as fold changes relative to controls using GAPDH (for lncRNAs) and U6 (for miRNAs) as endogenous controls. Statistical comparisons were performed using one-way ANOVA with post hoc testing. p < 0.05 was considered statistically significant; **p < 0.001.

Figure 1 shows the fold-change expression profiles of the molecular biomarkers under investigation, together with sclerostin. When comparing CKD and hemodialysis patients with controls, all measured parameters, such as *lncRNA H19*, *lncRNA MEG3*, *lncRNA MIAT*, *miR-135a*, *miR-29a*, and serum sclerostin, were significantly higher (*p* < 0.001). Additionally, each marker demonstrated a remarkable increase in hemodialysis patients compared to CKD patients (*p*-value <0.001 for each).

### 3.3 Diagnostic performance of the investigated biomarkers

**FIGURE 2:**
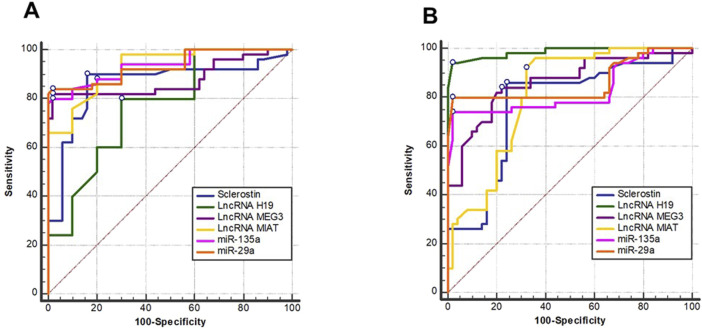
Receiver operating characteristics (ROC) curve illustrating the diagnostic value of sclerostin, lncRNA H19, lncRNA MEG3, lncRNA MIAT, miR-135, and miR-29 in distinguishing **(A)** CKD from controls and **(B)** CKD from hemodialysis.

ROC analysis used to assess the diagnostic efficacy of the investigated biomarkers for CKD discrimination showed that *lncRNA MIAT*, *miR-135a*, and *miR-29a* displayed the highest diagnostic performance in distinguishing CKD patients from healthy controls, with the greatest AUC values (Table 3; Figure 2).

**TABLE 3:** Diagnostic performance of the parameters studied for distinguishing patients.

Variables	Sensitivity %	Specificity %	Cut-off	AUC	PPV %	NPV %	Accuracy %
Discriminating CKD from controls							
Sclerostin (pg/mL)	90	84	>560.8	0.751	84.9	89.4	74
LncRNA *H19*	80	70	>1.11	0.769	72.7	77.8	50
LncRNA*MEG3*	82	98	>1.38	0.878	97.6	84.5	80
LncRNA *MIAT*	88	80	>1.2754	0.927	81.5	87	68
miR-135a	80	98	>1.6506	0.936	97.6	83.1	78
miR-29a	84	98	>1.4031	0.933	97.7	86	82
Discriminating CKD from hemodialysis							
Sclerostin (pg/mL)	86	76	>669.7	0.67	78.2	84.4	62
LncRNA *H19*	94	98	>2.28	0.984	97.9	94.2	92
LncRNA*MEG3*	84	78	>2.55	0.862	79.2	83	62
LncRNA *MIAT*	92	68	>2.299	0.81	74.2	89.5	60
miR-135a	74	98	>4.7338	0.827	97.4	79	72
miR-29a	80	98	>6.0151	0.860	97.6	83.1	78

The table summarizes sensitivity, specificity, cutoff values, positive predictive value (PPV), negative predictive value (NPV), AUC, and diagnostic accuracy for each biomarker.

The strongest predictors for distinguishing hemodialysis patients from CKD patients were lncRNA *H19*, *MEG3*, and *miR-29a*, indicating their significance in capturing late-stage disease processes.

### 3.4 Multivariate regression analysis of risk factors for CKD and hemodialysis patients

Sclerostin, lncRNA *MEG3*, and lncRNA *MIAT* were found to be significant independent predictors of CKD in multivariate regression analysis, whereas lncRNA H19, lncRNA MEG3, and lncRNA MIAT remained significant predictors of hemodialysis (Table 4).

**TABLE 4:** Multivariate regression analysis to determine the independent predictors of CKD and hemodialysis.

CKD			
Characteristics	Beta-coefficient	95% CI	*p*-value
Sclerostin (pg/mL)	0.142	0.000 to 0.001	**0.033**
LncRNA *H19* (fold change)	0.092	−0.043 to 0.283	0.148
LncRNA *MEG3* (fold change)	0.306	0.125 to 0.287	**< 0.001**
LncRNA *MIAT* (fold change)	0.152	0.006 to 0.129	**0.033**
*miR-135a* (fold change)	0.219	−0.029 to 0.197	0.144
*miR-29a* (fold change)	0.230	−0.012 to 0.129	0.102
Hemodialysis			
Sclerostin (pg/mL)	0.098	0.000 to 0.001	0.060
LncRNA *H19* (fold change)	0.587	0.272 to 0.423	**< 0.001**
LncRNA*MEG3* (fold change)	0.248	0.060 to 0.150	**< 0.001**
LncRNA*MIAT* (fold change)	0.128	0.008 to 0.071	**0.015**
*miR-135a* (fold change)	0.125	−0.012 to 0.090	0.136
*miR-29a* (fold change)	−0.023	−0.041 to 0.031	0.778

This table presents β-coefficients, 95% confidence intervals, and *p*-values from multivariate logistic regression models evaluating independent associations between biomarkers and CKD or hemodialysis status.

These results support the biological significance of these lncRNAs as molecular markers of disease severity, in line with research indicating that they become activated as renal injury worsens.

### 3.5 Correlations between molecular biomarkers and biochemical laboratory variables

The levels of sclerostin, lncRNA H19, lncRNA MEG3, lncRNA MIAT, miR-135, and miR-29 were all significantly positively correlated with one another. In addition, they were significantly positively correlated with levels of creatinine, urea, and phosphorus (Table 5), suggesting that these molecules may be co-regulated within common biological networks. Additionally, all markers showed negative correlations with eGFR and calcium.

**TABLE 5:** Correlation studies between sclerostin, lncRNA *H19*, lncRNA*MEG3*, lncRNA *MIAT*, miR-135, miR-29, and laboratory data in study participant**s**.

Variables		Sclerostin	LncRNA *H19*	LncRNA*MEG3*	LncRNA *MIAT*	miR-135	miR-29
Sclerostin							
LncRNA*H19*	r P	0.558*** <0.001					
LncRNA*MEG3*	r P	0.551*** <0.001	0.655*** **< 0.001**				
LncRNA *MIAT*	r P	0.591*** <0.001	0.763*** **< 0.001**	0.664*** **< 0.001**			
miR-135a	r P	0. 688*** **< 0.001**	0.790*** **< 0.001**	0.631*** <0.001	0.754*** **< 0.001**		
miR-29a	r P	0.691*** <0.001	0.831*** **< 0.001**	0.656*** **< 0.001**	0.758*** **< 0.001**	0.952*** **< 0.001**	
Creatinine	R P	0.693*** **< 0.001**	0.798*** **< 0.001**	0.731*** **< 0.001**	0.786*** **< 0.001**	0.789*** **< 0.001**	0.808*** **< 0.001**
Urea	R P	0.656*** **< 0.001**	0.654*** <0.001	0.674*** **< 0.001**	0.702*** **< 0.001**	0.747*** **< 0.001**	0.766*** **< 0.001**
eGFR	R P	−0.692*** **< 0.001**	−0.800*** **< 0.001**	−0.762*** **< 0.001**	−0.736*** **< 0.001**	−0.785*** **< 0.001**	−0.803*** **< 0.001**
Calcium	R P	−0.365*** **< 0.001**	−0.411*** <0.001	−0.471*** **< 0.001**	−0.449*** **< 0.001**	−0.383*** <0.001	−0.395*** <0.001
Phosphorus	R P	0.523*** **< 0.001**	0.561*** **< 0.001**	0.502*** **< 0.001**	0.607*** <0.001	0.592*** <0.001	0.585*** **< 0.001**

Spearman correlation analysis was used to examine associations among sclerostin, lncRNAs, microRNAs, and biochemical markers. Abbreviations: r, Spearman correlation coefficient; *** *p* < 0.001 (highly significant).

## 4 Discussion

New molecular pathways and traditional biochemical parameter alterations interact intricately to drive the development of CKD and its complications in hemodialysis patients. Sclerostin and non-coding RNAs are two examples of non-traditional biomarkers that are useful in identifying early and mechanistic aspects of renal pathology. In this regard, the current study examined the expression of two microRNAs (*miR-135a and miR-29a*) and three lncRNAs (*H19, MEG3, and MIAT*), together with serum sclerostin levels, across hemodialysis and CKD stages compared to healthy controls. The overall pattern highlights the multifactorial nature of CKD progression and suggests that these molecules may be useful as biomarkers that also provide mechanistic insights.

The results of the present study follow the typical biochemical decline in renal function, with increased serum creatinine, urea, and phosphorus and decreased calcium, hemoglobin, and eGFR in patients with CKD and hemodialysis patients compared with controls, indicating worsening renal function and the systemic inflammatory process of CKD and hemodialysis (Obi et al., 2024). These results are associated with the established development of mineral bone disorder and anemia, which are associated with CKD (Izzo et al., 2024). The inflammatory environment of the hemodialysis patients, indicated by high levels of WBCs, can negatively influence the role of oxidative stress and lead to rapid renal fibrosis (Wang et al 2025). The impaired concentration of calcium, hemoglobin, platelets, and eGFR among CKD patients and, even more, among hemodialysis patients, once again proves the fact of complications that accompany the development of renal dysfunction, along with the severity of the disease (Salera et al.,2025).

One of our study’s main findings is the marked elevation of the Wnt pathway inhibitor, sclerostin, in both CKD and hemodialysis patients; its levels also correlate with CKD progression. This is consistent with its role in vascular calcification and bone metabolism dysregulation (Petrović et al.,2024; Xing et al., 2024); according to the correlation between CKD progression and sclerostin, sclerostin may be involved in both skeletal abnormalities and renal tissue remodeling (Lin et al., 2023). Its function as an integrated indicator of mineral, vascular, and renal interactions is further supported by its strong correlations with traditional biochemical markers.

All examined lncRNAs, including *H19*, *MEG3*, *MIAT*, *miR-135a*, and *miR-29a*, proved significant upregulation in CKD and even more so in hemodialysis patients, in tandem with the increase in serum sclerostin levels. These steady increases highlight their possible role in the molecular cascades driving the advancement of CKD. The lncRNAs analyzed here are associated with processes essential to the pathophysiology of CKD, including fibrosis, apoptosis, oxidative stress, endothelial dysfunction, and epithelial–mesenchymal transition (EMT). *H19* is connected to inflammatory and pro-fibrotic signaling; *MEG3* has been linked to *TGF-β*-mediated fibrosis and dysregulated apoptosis (Meng et al., 2016; Hussain et al., 2023; Jiang, 2025); and *MIAT* has been linked to endothelial damage and vascular problems (Wang et al., 2024). Their usefulness as sensitive markers of renal structural and functional decline is supported by their progressive elevation across disease stages in our study. The simultaneous overexpression of *miR-135a* and *miR-29a* simultaneously has also been shown to contribute to underlying pathophysiological mechanisms. The *miR-29* family, in particular *miR-29a*, has a basic anti-fibrotic effect on renal disease by directing the downregulation of extracellular matrix genes, such as collagens and fibronectin; it has been experimentally downregulated to stimulate renal fibrosis in CKD (Qin et al., 2011). However, although *miR-29a* can suppress extracellular matrix proteins and is an incredibly crucial anti-fibrotic factor, malfunctioning of *miR-135a* is associated with the involvement of inflammatory and fibrotic processes (Huang et al., 2025). The competitive endogenous RNA (ceRNA) networks of lncRNAs control the accessibility of miRNAs to increase the fibrotic and inflammatory responses. This may be the reason that led to concerted lncRNA and microRNA upregulation in this study.

Our studied biomarkers can potentially be useful diagnostics as the ROC analysis supports this idea. LncRNA *MIAT*, *miR-135a*, and *miR-29a* were found to be excellent at differentiating CKD and controls and thus useful in early detection, when eGFR and creatinine might be under-representative of the underlying incipient molecular injury. Similarly, *H19, MEG3*, and *MIAT* performed better with the ability to distinguish between hemodialysis and non-dialysis CKD patients, indicating that these parameters can be used as tools to identify the transitions of ESRD. These findings imply that combining molecular biomarkers with traditional clinical parameters could improve risk assessment, guide management strategies, and enhance diagnostic accuracy.

The non-coding RNA biomarkers measured in this study appear to reflect earlier and more mechanism-focused alterations in renal injury compared with standard CKD indicators, including creatinine, urea, and eGFR. Conventional markers start increasing when a significant amount of nephron failure has occurred, but lncRNAs and miRNAs react to initial molecular disruptions, including fibrosis, cell death, EMT, and Wnt/TGF-b pathway imbalances. This may explain why some ncRNAs were highly discriminative in the ROC analysis, even during the earlier stages of CKD, when creatinine and eGFR were still at borderline or only slightly modified levels. In addition, the relationships that we have found between these ncRNAs and disease severity indices indicate that they reflect continuous pathological events, rather than a reduction in filtration capacity. Hence, the molecular biomarkers used are complementary to the clinical markers as they provide some mechanistic understanding and even early warning signs that are not available in conventional tests.

Correlation analysis further supports the biological plausibility of these biomarkers. Similarity in regulatory pathways is indicated by the strong positive correlations between sclerostin and lncRNAs, microRNAs, and biochemical indices such as creatinine and phosphorus and by negative correlations with eGFR and calcium. They may consist of oxidative stress, vascular calcification, Wnt/b-catenin suppression, *TGF-b/Smad*-mediated fibrosis, and chronic low-grade inflammation—all of which are hallmarks of CKD progression (Tang et al., 2022). This inter-relationship of behavior is an indication of a more system-wide dysregulation, rather than isolated molecular abnormalities.

Multivariate regression analysis revealed that sclerostin, *MEG3*, and *MIAT* were independent predictors of CKD, while *H19*, *MEG3*, and *MIAT* were strong predictors of hemodialysis status. These results indicate that sclerostin and lncRNAs are important potential clinical tools for assessing disease severity. These markers can be actively involved in pathological development and the indication of persistent damage since they are involved mechanistically in fibrosis, EMT, and vascular dysfunction (Hou and Yi, 2024). Since such specific inhibitions as antisense oligonucleotides against MEG3 have already been shown to reduce fibrosis and vascular calcification in preclinical studies, their possible clinical utility is also of interest (Wu et al., 2024).

Collectively, these findings indicate that sclerostin and the tested lncRNAs and microRNAs are active markers in the biology of CKD. Their presence in the extracellular matrix deposition, oxidative stress, endothelial dysfunction, inflammation, and vascular complications is consistent with the multifaceted character of CKD pathogenesis. Hemodialysis-related oxidative stress could also amplify these pathways, which may explain why dialysis patients showed upregulated expression profiles for all studied biomarkers. These molecular biomarkers correlate with traditional biochemical markers, demonstrating the importance of using both clinical and molecular diagnostics to gain a comprehensive insight into the complexity of chronic kidney disease.

In this study, we conclude that a panel of promising molecular biomarkers can be identified for use in CKD and hemodialysis patients to detect the disease at its earliest stage and evaluate the risk of disease progression. Although these are preliminary findings, our data suggest that a combined biomarker signature should be considered because this can lead to an improved diagnostic accuracy compared to single biomarkers. These findings may enhance our understanding of CKD pathophysiology, but longitudinal and interventional studies are needed to determine their prognostic and therapeutic value.

Future molecular profiling-based clinical decision-making could support the use of more personalized approaches to the treatment of CKD and its complications.

### 4.1 Summary and recommendations

In summary, our research clarifies the diagnostic and prognostic potential of circulating biomarkers in CKD and hemodialysis patients, including lncRNAs (*H19*, *MEG3*, and *MIAT*) and miRNAs (*miR-135a* and *miR-29a*). The results support additional studies to confirm these biomarkers in bigger cohorts and examine the mechanisms underlying renal pathophysiology.

## 5 Limitations

We acknowledge several limitations in this study. First, the moderate sample size and the lack of CKD staging restrict the ability to evaluate stage-specific alterations in circulating parameters. Finally, the absence of an external validation cohort limits the assessment of biomarker performance in other populations. Future studies with larger sample sizes and stage-specific assessments are warranted to confirm and extend our findings.

## Data Availability

The datasets presented in this study can be found in online repositories. The names of the repository/repositories and accession number(s) can be found in the article/supplementary material.
